# Sleep EEG signatures in mouse models of 15q11.2-13.1 duplication (Dup15q) syndrome

**DOI:** 10.1186/s11689-024-09556-7

**Published:** 2024-07-16

**Authors:** Vidya Saravanapandian, Melika Madani, India Nichols, Scott Vincent, Mary Dover, Dante Dikeman, Benjamin D. Philpot, Toru Takumi, Christopher S. Colwell, Shafali Jeste, Ketema N. Paul, Peyman Golshani

**Affiliations:** 1 Department of Neurology and Semel Institute for Neuroscience, David Geffen School of Medicine, 710 Westwood Plaza, Los Angeles, CA 90095 USA; 2 Department of Integrative Biology and Physiology, University of California Los Angeles, Los Angeles, CA 90095 USA; 3 Department of Biology, Spelman College, 350 Spelman Lane, Atlanta, GA 30314 USA; 4 Neuroscience Center, Department of Cell Biology and Physiology, and the Carolina Institute for Developmental Disabilities, UNC-Chapel Hill, NC 27599 USA; 5 Kobe University School of Medicine, Chuo, Kobe, 650-0017 Japan; 6 Department of Psychiatry & Biobehavioral Sciences, University of California Los Angeles, Los Angeles, CA 90095 USA; 7 Children’s Hospital Los Angeles, 4650 Sunset Blvd, MS 82, Los Angeles, CA 90027 USA; 8 West Los Angeles VA Medical Center, 11301 Wilshire Blvd, Los Angeles, CA 90073 USA

**Keywords:** Dup15q syndrome, Autism, Biomarkers, Sleep, EEG, GABA, UBE3A, Neurodevelopmental disorders

## Abstract

**Background:**

Sleep disturbances are a prevalent and complex comorbidity in neurodevelopmental disorders (NDDs). Dup15q syndrome (duplications of 15q11.2-13.1) is a genetic disorder highly penetrant for NDDs such as autism and intellectual disability and it is frequently accompanied by significant disruptions in sleep patterns. The 15q critical region harbors genes crucial for brain development, notably UBE3A and a cluster of gamma-aminobutyric acid type A receptor (GABA_A_R) genes. We previously described an electrophysiological biomarker of the syndrome, marked by heightened beta oscillations (12-30 Hz) in individuals with Dup15q syndrome, akin to electroencephalogram (EEG) alterations induced by allosteric modulation of GABA_A_Rs. Those with Dup15q syndrome exhibited increased beta oscillations during the awake resting state and during sleep, and they showed profoundly abnormal NREM sleep. This study aims to assess the translational validity of these EEG signatures and to delve into their neurobiological underpinnings by quantifying sleep physiology in chromosome-engineered mice with maternal (matDp/ + mice) or paternal (patDp/ + mice) inheritance of the full 15q11.2-13.1-equivalent duplication, and mice with duplication of just the UBE3A gene (*Ube3a* overexpression mice; Ube3a OE mice) and comparing the sleep metrics with their respective wildtype (WT) littermate controls.

**Methods:**

We collected 48-h EEG/EMG recordings from 35 (23 male, 12 female) 12–24-week-old matDp/ + , patDp/ + , Ube3a OE mice, and their WT littermate controls. We quantified baseline sleep, sleep fragmentation, spectral power dynamics during sleep states, and recovery following sleep deprivation. Within each group, distinctions between Dup15q mutant mice and WT littermate controls were evaluated using analysis of variance (ANOVA) and student’s t-test. The impact of genotype and time was discerned through repeated measures ANOVA, and significance was established at *p* < 0.05.

**Results:**

Our study revealed that across brain states, matDp/ + mice mirrored the elevated beta oscillation phenotype observed in clinical EEGs from individuals with Dup15q syndrome. Time to sleep onset after light onset was significantly reduced in matDp/ + and Ube3a OE mice. However, NREM sleep between Dup15q mutant and WT littermate mice remained unaltered, suggesting a divergence from the clinical presentation in humans. Additionally, while increased beta oscillations persisted in matDp/ + mice after 6-h of sleep deprivation, recovery NREM sleep remained unaltered in all groups, thus suggesting that these mice exhibit resilience in the fundamental processes governing sleep-wake regulation.

**Conclusions:**

Quantification of mechanistic and translatable EEG biomarkers is essential for advancing our understanding of NDDs and their underlying pathophysiology. Our study of sleep physiology in the Dup15q mice underscores that the beta EEG biomarker has strong translational validity, thus opening the door for pre-clinical studies of putative drug targets, using the biomarker as a translational measure of drug-target engagement. The unaltered NREM sleep may be due to inherent differences in neurobiology between mice and humans. These nuanced distinctions highlight the complexity of sleep disruptions in Dup15q syndrome and emphasize the need for a comprehensive understanding that encompasses both shared and distinct features between murine models and clinical populations.

## Background

Sleep disturbances represent a pervasive yet often overlooked aspect of neurodevelopmental disorders (NDDs) [1, 2], contributing to the complex clinical presentation of individuals affected by these conditions [3–5]. The frequent co-occurrence of sleep disturbances and NDDs underscores the critical role of healthy sleep in cognitive, emotional, and physical well-being. However, the intricate interplay between genetic, neural, and environmental factors that govern sleep in people with NDDs is poorly understood. This study explores the multifaceted landscape of sleep in NDDs, with a focus on Dup15q syndrome, a rare genetic disorder with duplications of the chromosome 15q11.2-13.1 region. Dup15q syndrome is characterized by autism spectrum disorder (ASD), intellectual disability (ID), hypotonia and related motor impairment, epilepsy, and sleep issues [6–10]. Despite the high prevalence of sleep disturbances reported in individuals with Dup15q syndrome [7, 10], the neurobiological mechanisms governing these disturbances remain unexplored. Previous investigations into sleep patterns in Dup15q syndrome, conducted primarily in clinical populations, have highlighted alterations in sleep architecture, spectral dynamics, and other sleep microstructures [6, 10]. Specifically, on electroencephalography (EEG) recordings collected from individuals with Dup15q syndrome, beta band oscillations (12-30Hz) were elevated during the awake resting state [11–13] and during sleep [6, 10]. Furthermore, Non-rapid eye movement sleep (NREM) sleep was profoundly abnormal with reduced sleep spindles and slow wave sleep [6, 10]. Yet, the involvement of specific gene duplication in these physiological alterations remains unknown.

Mouse models provide a controlled environment to examine the underlying mechanisms and pathways associated with this syndrome. The potential to identify a clinical biomarker in mice can provide better assessments of treatment efficacy and aid in the development of targeted interventions, contributing to the overall success of clinical trials. Transgenic mouse models, including those with maternal (matDp/ + mice) or paternal (patDp/ + mice) inheritance of the full 15q11.2–13.1-equivalent duplication region [14] and those overexpressing just *Ube3a* (Ube3a OE mice) in the region [15], provide a unique opportunity to dissect the specific neurobiological alterations associated with Dup15q syndrome.

In humans, maternally derived duplications of chromosome 15q11.2-13.1 collectively present one of the most common copy number variants associated with NDDs [16, 17]. Clinical symptom severity has shown to increase with the number of 15q11.2-13.1 copies, thus resulting in individuals with isodicentric triplication (having 3 copies of the 15q region) having worse symptoms compared to those with interstitial duplications (having 2 copies of the 15q region) [8, 18, 19]. Several genes in the 15q critical region are overexpressed. Most notably, UBE3A, a gene that encodes the ubiquitin protein ligase, is imprinted in neurons [20, 21] and regulates synaptic function [22–25]. In addition, a cluster of gamma-aminobutyric acid type A receptor (GABA_A_R) genes, GABRB_3_, GABRA_5_, and GABRG_3_, which encode the β3, α5 and γ3 receptor subunits, respectively are also duplicated [26, 27]. Maternal inheritance of UBE3A gene duplications in patients has been linked to developmental delay [28], and patDp/ + mice exhibit ASD-like phenotypes like those seen in humans [14]. However, findings from *Ube3a* overexpression mouse models have been contradictory based on differences in mouse model design. Some studies have shown that *Ube3a* overexpression mice exhibit core ASD features, learning deficits, anxiety-like behavior, and reduced seizure threshold [29], while others, despite the overexpression of *Ube3a*, have much more subtle findings [15].

Studies have shown that GABA_A_R modulators such as benzodiazepines induce patterns of beta oscillations, like that seen in children with Dup15q syndrome [30–35]. Moreover, the loss of neuronal expression of maternally inherited UBE3A gene due to deletions in the 15q critical region results in Angelman syndrome (AS) [36, 37], a disorder whose clinical symptoms overlap with that of Dup15q syndrome, namely the presence of ID, ASD, and epilepsy. Taken together, quantification of sleep physiology in mouse models of Dup15q syndrome, particularly those with and without the GABA_A_R genes, will help elucidate the effects of putative genes in the 15q region on disrupted sleep. Therefore, in this study, we collected 48-h EEG/EMG recordings from matDp/ + , patDp/ + , and Ube3a OE mouse models and their wildtype (WT) littermate controls, and quantified the following sleep parameters: baseline sleep, sleep fragmentation, spectral dynamics during different sleep states, and recovery sleep following sleep deprivation. We found that matDp/ + mice exhibited elevated beta oscillations similar to those seen in the clinical population with Dup15q syndrome. Additionally, matDp/ + and Ube3a OE mice showed significantly reduced time to sleep onset. However, contradictory to expectations, none of the three mice groups showed alterations in NREM sleep and all were able to recover from sleep deprivation. This study provides the first quantitative assessment of sleep physiology in Dup15q mice and validates the translatability of the beta EEG phenotype, thus making it a robust preclinical biomarker that can be utilized as an outcome measure in clinical trials.

## Methods

### Animals

**Fig. 1:**
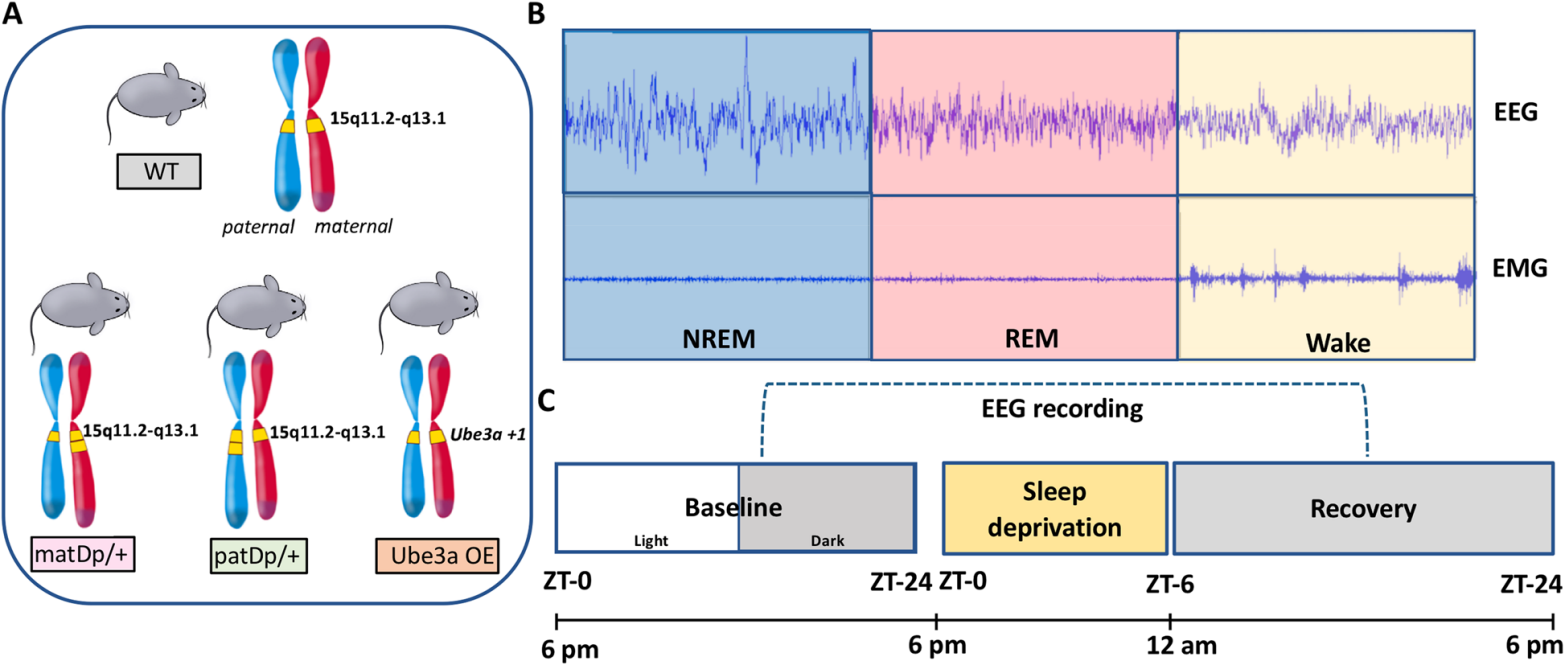
Mice groups in the study and recording timeline. Three Dup15q mutant groups included in the study: matDp/ + , patDp/ + , and Ube3a OE (**A**) were compared with each of their wildtype (WT) littermate controls. Representative example of a brief period of EEG and EMG signal from NREM, REM and Wake states (**B**). Schematic timeline of EEG/EMG recording, which includes 24 h of baseline, 6 h of sleep deprivation and 18 h of spontaneous recovery sleep (**C**)

All experiments were conducted per National Institute of Health (NIH) guidelines and with the approval of the Chancellor’s Animal Research Committee of the University of California, Los Angeles. The study was conducted on 47 (27 male, 19 female) 12-24 week-old mice and included the following genetically modified mice groups and wildtype littermate controls (Fig. 1A): 1) matDp/ + mice (which have a 6.3 Mb duplication of mouse chromosome 7 mirroring the human chromosome 15q11.2-13.1 duplication [14], 2) patDp/ + mice with the same duplication at matDp/ + but inherited paternally [14], and 3) Ube3a OE mice (line EO6), which carry one extra copy of UBE3A gene (*Ube3a*^+*1*^), resulting in overexpression of *Ube3a* [15]. matDp/ + and patDp/ + mice were obtained from Dr. Takumi’s laboratory through Riken Bioresource Research Center. Ube3a OE mice were obtained directly from Dr. Ben Philpot’s laboratory. In each group, mutant males were crossed with wildtype females of the same genetic background to generate both experimental and control offspring in the same litter. Animals were 12–24 weeks old at the time of surgery and were maintained on a 12-h light and 12-h dark cycle with freely available food and water. Animals that showed severe artifacts in their recordings were excluded from analysis. After exclusion, a final group of 35 mice (matDp/ + : 9 mutants, 8 WT; patDp/ + : 6 mutants and 4 WT, and Ube3a OE: 5 mutants and 3 WT) were included in the analysis. Table 1 includes the sex breakdown in each group.

**Table 1:** Mouse population characteristics in the study

Genotype		Sex		Total
		Male	Female	
matDp/ +	Mutant	6	3	9
	WT	6	2	8
patDp/ +	Mutant	3	3	6
	WT	2	2	4
Ube3a	Mutant	5	0	5
	WT	3	0	3

### Surgery

Animals were anesthetized with isoflurane (3% induction, 1.5% maintenance) using a precision vaporizer and maintained on a heated pad throughout the procedure. All mice received a subcutaneous injection of buprenorphine (3.25 mg/kg) 30 min prior to the surgery for analgesia. Anesthetized mice were implanted with 4 EEG and 2 EMG electrodes. A prefabricated head mount (Pinnacle Technologies, KS) was used to position three stainless-steel epidural screw electrodes. The first electrode (frontal—located over the frontal cortex) was placed 1.5 mm anterior to bregma and 1.5 mm lateral to the central suture, whereas the second two electrodes (interparietal—located over the visual cortex and common reference) were placed 2.5 mm posterior to bregma and 1.5 mm on either side of the central suture. The resulting two leads (frontal-interparietal and interparietal-interparietal) were references contralaterally. A fourth screw served as a ground. Electrical continuity between the screw electrode and head mount was aided by silver epoxy. EMG activity was monitored using stainless-steel Teflon-coated wires that were inserted bilaterally into the nuchal muscle. The head mount (integrated 2 X 3 pin grid array) was secured to the skull with dental acrylic. Mice were allowed to recover for at least 14 days before sleep recording.

### EEG/EMG recording

After one week of recovery from surgery, implanted mice were moved to sound-proof and light-controlled recording chambers (Pinnacle Technology, Inc.). Mice were tethered to a preamplifier (8202-SL, Pinnacle Technology, Inc.) and a low-resistance commutator (8204, Pinnacle Technology, Inc.), and were habituated to the tethered recording system for one week. Mice were able to move freely around their cages during the habituation and polysomnographic recording periods. EEG/EMG recordings started at zeitgeber time (ZT) zero. For baseline recording, animals were recorded for 24 h, with 12 h of light on (Light) and 12 h of light off (Dark) (Fig. 1C). Immediately following the 24-h baseline recording, mice were subjected to a 6-h total sleep deprivation by a gentle handling procedure performed by experts blinded to the experimental conditions starting at ZT 0 followed by 18 h of recovery sleep (Fig. 1C). Recordings were low pass filtered with a 40-Hz cutoff and digitized at a 400-Hz sampling rate using Sirenia Acquisition software and analyzed using the Sirenia Sleep Pro software (Pinnacle Technology, Inc.).

### Signal processing and analysis

Recordings were manually inspected and scored into 10-s epochs and brain states were assigned based on the frequency and amplitude signatures of consecutive epoch windows as either wake (low-amplitude desynchronized EEG and high-amplitude EMG activity), NREM (synchronized EEG with high-amplitude mixed-frequency EEG activity and low-amplitude EMG activity), or rapid eye movement sleep (REM sleep) (which is low-amplitude EEG with prominent theta activity with 6-9 Hz EEG and low-amplitude EMG activity) (Fig. 1B). Percentage of time spent in each brain state was recorded. The duration of time taken for animals to accumulate at least one bout of NREM sleep (20 s or longer) following the beginning of their inactive (light) phase was recorded as time to sleep onset. Based on variations in bout durations during wakefulness, short wakeful bouts (bouts less than 1 min) were defined as brief arousals, and long wakeful bouts (bouts longer than 2 min) were defined as stable wakefulness.

Each recording was scored by two scoring experts blinded to genotype and experimental conditions and the agreement between the two scorers was reviewed. Power spectral analysis was performed by applying a fast Fourier transform (FFT) to raw EEG waveforms. Delta, theta, and beta power were measured as spectral power in the 0.5-4 Hz, 4-8 Hz, and 12-30 Hz frequency range respectively, and each expressed as a percentage of total spectral power in the EEG signal (0.5-40 Hz). Similar to the continuous baseline recordings, recordings during sleep deprivation and during sleep recovery were evaluated, and the total time spent in NREM sleep, the number of arousals and time to sleep onset were quantified. Data were analyzed using GraphPad Prism, version 8.1.2 (227). Data were analyzed using analysis of variance (ANOVA) and student’s t-test to compare and detect differences between Dup15q mutant mice and WT littermate controls in each group. A repeated measures ANOVA was used to detect the effect of genotype and time. Significance was set at *p* < 0.05.

## Results

### Evaluation of 24-h baseline EEG recording in Dup15q syndrome

**Fig. 2:**
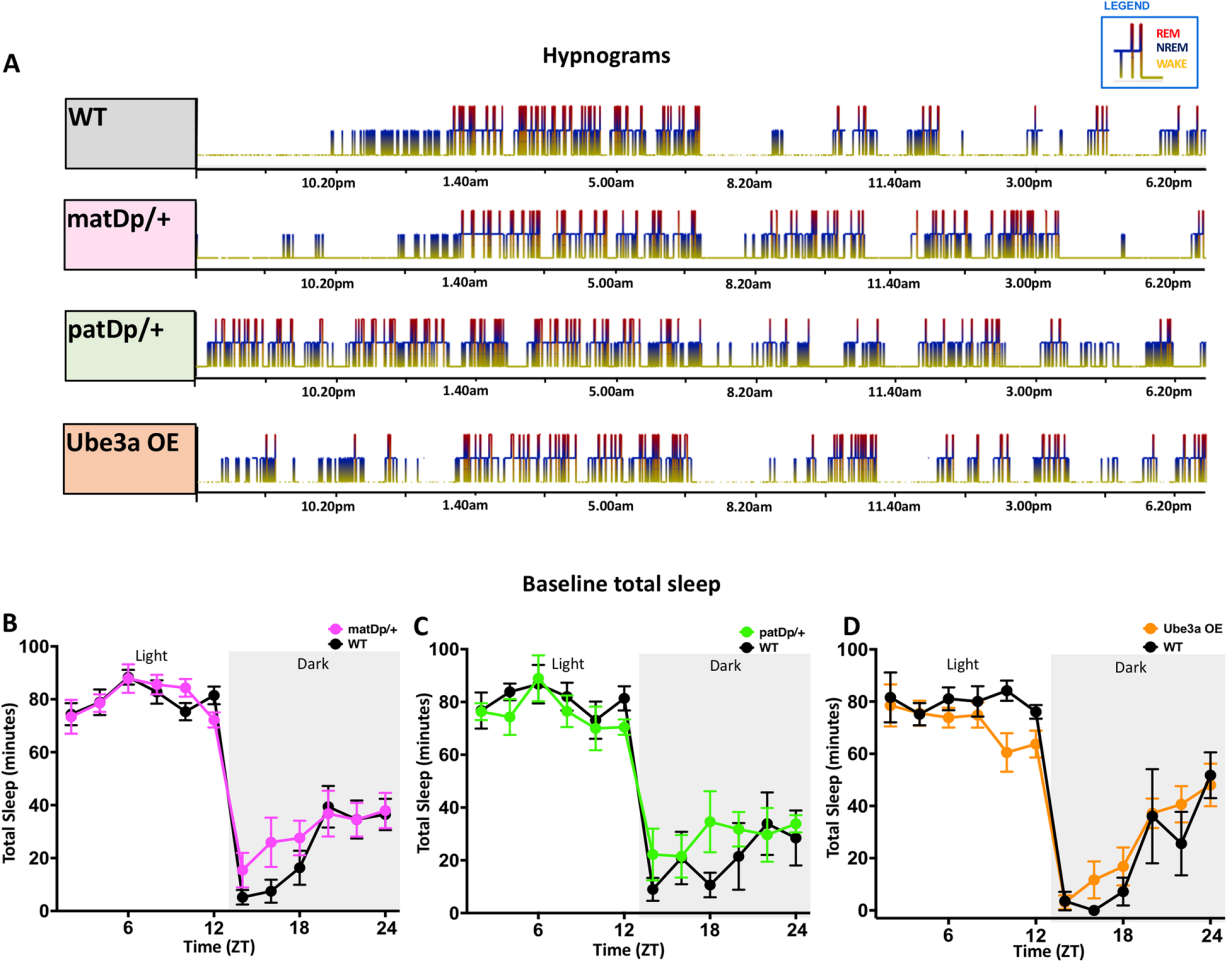
Overview of sleep architecture during 24-h baseline recording. Representative hypnograms of WT, matDp/ + , patDp/ + , and Ube3a OE mice (**A**) during 24-h baseline recordings. Temporal pattern of sleep (both NREM and REM) during 24-h baseline recordings in matDp/ + (**B**), patDp/ + (**C**), and Ube3a OE (**D**) models of Dup15q mice compared to their WT littermate controls. In panels B-D, the background shading represents the dark period

Humans with Dup15q syndrome have severe disruptions in NREM sleep. To determine whether these sleep disruptions can be modeled in mice and which genes could potentially contribute, we performed 24-h baseline EEG recordings in mice with maternal inheritance of the full duplication (matDp/ +) [14], paternal inheritance of the full duplication (patDp/ +) [14], or duplications solely of *Ube3a* (one extra copy of the gene, *Ube3a*^+*1*^) (Ube3a OE) [15] and littermate controls. To measure the ability to recover from sleep loss, we examined how these mice responded to a 6-h sleep deprivation followed by an 18-h recovery period. We quantified the amount of NREM, REM, and wakefulness. Representative sleep hypnograms collected from matDp/ + , patDp/ + , and Ube3a OE mouse models, during the 24-h continuous baseline EEG recordings showed the presence of all sleep stages and no obvious differences in the ultradian sleep cycle, compared to that of a wildtype (WT) littermate control from the matDp/ + group (Fig. 2A). Examination of sleep EEG recordings using 2-h bins revealed there were no significant differences in total sleep time (including time spent in both NREM and REM sleep) during the undisturbed 12-h light (resting period) and 12-h dark (active period) phase recordings in matDp/ + , F_(11, 165)_ = 1.051, *P* = 0.4048 (Fig. 2B), patDp/ + , _(11, 66)_ = 0.9327, *P* = 0.5153 (Fig. 2C), and Ube3a OE, F_(11, 66)_ = 1.173, *P* = 0.3225 (Fig. 2D) models of Dup15q mice, compared to their WT littermate controls.

### Quantification of NREM sleep, REM sleep, and sleep fragmentation

**Fig. 3:**
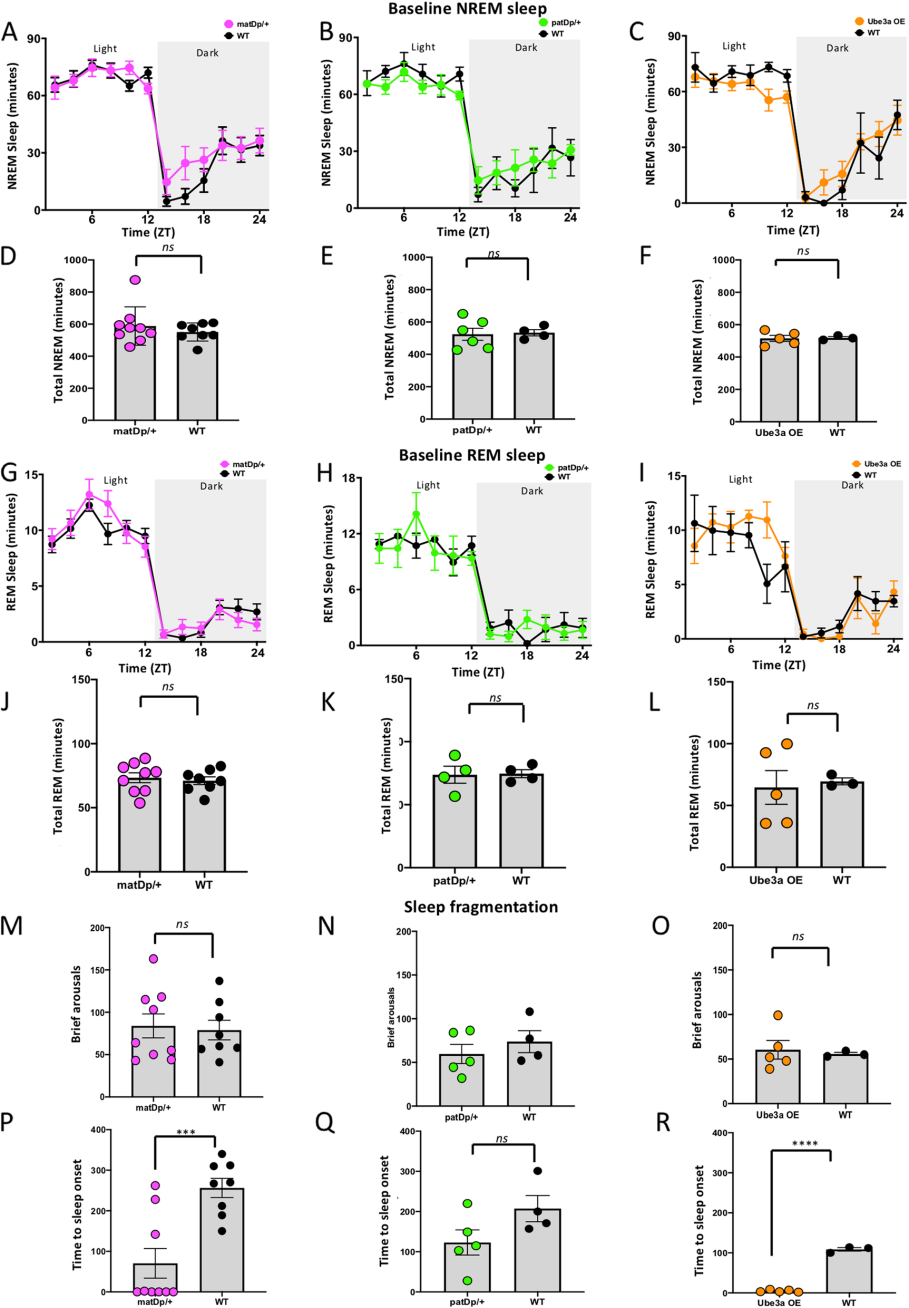
Temporal pattern of the different sleep stages in Dup15q mutant mice and controls. Temporal pattern of NREM sleep in 24-h baseline continuous sleep recording in matDp/ + (**A**), patDp/ + (**B**), and Ube3a OE (**C**) mice compared to their littermate controls. Total NREM sleep time in matDp/ + (**D**), patDp/ + (**E**), and Ube3a OE (**F**) mice. Temporal pattern of REM sleep in matDp/ + (**G**), patDp/ + (**H**), and Ube3a OE (**I**) mice compared to their littermate controls. Total REM sleep time in matDp/ + (**J**), patDp/ + (**K**), and Ube3a OE (**L**). Number of brief arousals (**M**–**O**) and time to sleep onset (**P**-**R**) compared between mutants and WT controls in the matDp/ + , patDp/ + , and Ube3a OE groups. In panels **A**-**C** and **G**-**I**, the background shading represents the dark period

We further analyzed how the duration of time spent in NREM sleep varied and how this related to the genotype of mice. The temporal pattern of NREM sleep analyzed using a two-way ANOVA with genotype and time as factors, showed no differences between Dup15q mutant mice: matDp/ + , F_(11, 165)_ = 1.210, *P* = 0.2837 (Fig. 3A); patDp/ + , F_(11, 88)_ = 0.7015, *P* = 0.7339 (Fig. 3B); Ube3a OE, F_(11, 66)_ = 1.028, *P* = 0.4326 (Fig. 3C), and their littermate controls. The total time spent in NREM sleep also showed no differences between the groups: matDp/ + , *P* = 0.4339, t = 0.8042, df = 15 (Fig. 3D); patDp/ + , *P* = 0.8483, t = 0.1976, df = 8 (Fig. 3E); Ube3a OE, *P* = 0.8963, t = 0.1360, df = 6 (Fig. 3F). We found similar results for REM sleep. We found no differences in the temporal pattern of REM sleep between Dup15q mutant mice and their littermate controls: matDp/ + , F_(11, 165)_ = 0.9631, *P* = 0.4821 (Fig. 3G); patDp/ + , F_(11, 66)_ = 0.8797, *P* = 0.5640 (Fig. 3H); Ube3a OE, F_(11, 66)_ = 1.566, *P* = 0.1299 (Fig. 3I). We found no differences in the total time spent in REM sleep between the groups: matDp/ + , *P* = 0.6487, t = 0.4648, df = 15 (Fig. 3J); patDp/ + , *P* = 0.9104, t = 0.1173, df = 6 (Fig. 3K); Ube3a OE, *P* = 0.7967, t = 0.2694, df = 6 (Fig. 3L). Table 2 includes analysis of sleep waveforms during baseline recording, separated by light and dark cycles.

**Table 2:** Analysis of sleep waveforms during baseline light and dark phase recordings using two-way ANOVA with genotype (group 1: matDp/ + , WT; group 2: patDp/ + , WT; group 3: Ube3a, WT) and time (2-h bins) as factors. Interactions reported for genotype x time. Significance was established at *p* < .05. In this and other tables significant values are indicated in bold

State	Genotype	Interactions			
		Light phase		Dark phase	
Total sleep (NREM + REM)	matDp/ +	F (5, 75) = 1.017	*P* = 0.4139	F (5, 75) = 1.054	*P* = 0.3928
	patDp/ +	F (5, 30) = 0.3068	*P* = 0.9050	F (5, 30) = 0.8254	*P* = 0.5416
	Ube3a	F (5, 30) = 1.458	*P* = 0.2329	F (5, 30) = 0.4067	*P* = 0.8403
NREM	matDp/ +	F (5, 75) = 1.228	*P* = 0.3047	F (5, 75) = 1.056	*P* = 0.3919
	patDp/ +	F (5, 40) = 0.5164	*P* = 0.7622	F (5, 40) = 0.5250	*P* = 0.7559
	Ube3a	F (5, 30) = 1.091	*P* = 0.3854	F (5, 30) = 0.3652	*P* = 0.8683
REM	matDp/ +	F (5, 75) = 1.167	*P* = 0.3333	F (5, 75) = 0.8687	*P* = 0.5063
	patDp/ +	F (5, 30) = 0.8655	*P* = 0.5156	F (5, 30) = 1.129	*P* = 0.3664
	Ube3a	F (5, 30) = 2.749	***P* = 0.0369**	F (5, 30) = 0.7148	*P* = 0.6173

We examined indices of sleep fragmentation during NREM sleep from the 24-h baseline EEG recordings. There were no differences in the number of brief arousals exhibited by Dup15q mice compared to their littermate controls in all three groups: matDp/ + , *P* = 0.7932, t = 0.2669, df = 15 (Fig. 3M); patDp/ + , *P* = 0.4222, t = 0.8523, df = 7 (Fig. 3N); Ube3a OE, *P* = 0.7467, t = 0.3382, df = 6 (Fig. 3O). However, matDp/ + and Ube3a OE mice exhibited significantly decreased time to sleep onset compared to their WT littermate controls: matDp/ + , *P* = 0.0009, t = 4.152, df = 15 (Fig. 3P); Ube3a OE, *P* < 0.0001, t = 28.31, df = 6 (Fig. 3R). No differences in time to sleep onset were seen in the patDp/ + group (*P* = 1.067, t = 1.850, df = 7) (Fig. 3Q). Therefore, there were no differences in the temporal patterns of NREM, REM sleep or wakefulness in Dup15q model animals, while matDp/ + and Ube3a OE mice showed reduced time to sleep onset compared to controls. This markedly differs from the sleep pattern found in humans with Dup15q where there is significant disruption in NREM sleep.

### Altered spectral power dynamics in Dup15q mice

**Fig. 4:**
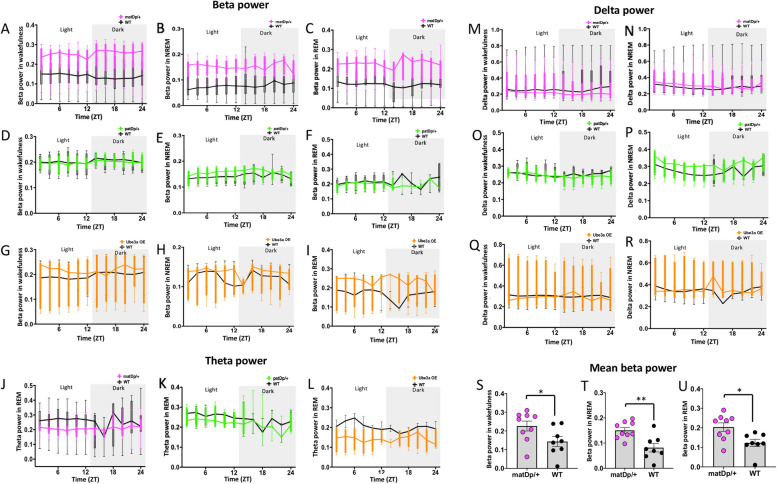
Spectral power dynamics during wakefulness and sleep. Beta power dynamics during wakefulness, NREM, and REM sleep in matDp/ + (**A**-**C**), patDp/ + (**D**-**F**), and Ube3a OE (**G**-**I**) groups compared to WT littermate controls. Delta power dynamics during wakefulness and NREM sleep in matDp/ + (**J**, **M**), patDp/ + (**K**, **N**), and Ube3a OE (**L**, **O**) groups compared to WT littermate controls. Theta power dynamics during REM sleep in matDp/ + (**P**), patDp/ + (**Q**), and Ube3a OE (**R**) groups compared to WT littermate controls. Mean beta power calculated across the 24-h recording compared between matDp/ + and WT littermate controls during wakefulness (**S**), NREM (**T**), and REM (**U**) sleep

Humans with Dup15q show elevated beta power and reduced delta power during wakefulness and during sleep [10, 12, 13, 38]. To determine whether these EEG biomarkers are also disrupted in the animal models, we analyzed spectral power of the EEG during wakefulness and NREM, and REM sleep. Power spectral analysis of 24-h continuous EEG recordings revealed that mutant mice in the matDp/ + group had elevated beta power compared to their littermate controls (Fig. 4 A-C). Mean beta power calculated across the recording showed significantly elevated beta power in the matDp/ + group across sleep–wake states, wakefulness, *P* = 0.0455, t = 2.181, df = 15 (Fig. 4S); NREM, *P* = 0.0032, t = 3.504, df = 15 (Fig. 4T), and REM sleep, *P* = 0.0132, t = 2.808, df = 15 (Fig. 4U). Beta power, however, did not differ between mutant mice and WT controls in both patDp/ + : wakefulness, F_(11, 66)_ = 0.4612, *P* = 0.9204 (Fig. 4D); NREM, F_(11, 60)_ = 0.6485, *P* = 0.7799 (Fig. 4E); REM, F_(11, 53)_ = 0.8368, *P* = 0.6048 (Fig. 4F), and the Ube3a OE: wakefulness, F_(11, 66)_ = 0.8359, *P* = 0.6053 (Fig. 4G); NREM, F_(11, 60)_ = 0.6485, *P* = 0.7799 (Fig. 4H); REM, F_(11, 57)_ = 1.254, *P* = 0.2746 (Fig. 4I) groups. Theta power during REM sleep did not differ between Dup15q mice and WT controls in all three groups: matDp/ + , F_(11, 134)_ = 0.9563, *P* = 0.4894 (Fig. 4J); patDp/ + , F_(11, 55)_ = 0.3265, *P* = 0.9765 (Fig. 4K); Ube3a OE, F_(11, 58)_ = 0.208, *P* = 0.9865 (Fig. 4L). Delta power did not differ in all three groups either during wakefulness: matDp/ + , F_(11, 163)_ = 1.942, *P* = 0.0376 (Fig. 4M); patDp/ + , F_(11, 66)_ = 0.4920, *P* = 0.9018 (Fig. 4O); Ube3a OE, F_(11, 66)_ = 1.058, *P* = 0.4080 (Fig. 4Q), or during NREM sleep: matDp/ + , F_(11, 162)_ = 1.264, *P* = 0.2495 (Fig. 4N); patDp/ + , F_(11, 64)_ = 0.3663, *P* = 0.9645 (Fig. 4P); Ube3a OE, F_(11, 59)_ = 0.8547, *P* = 0.5878 (Fig. 4R). Therefore, matDp/ + mice, but not the other mouse models, show elevated beta power across different sleep and wake stages like humans, but none of the models show any changes in delta power, indicating that mice can recapitulate some of the EEG biomarker changes in humans with Dup15q syndrome. Table 3 includes analysis of power spectral dynamics during baseline recording, separated by light and dark cycles.

**Table 3:** Analysis of power spectral dynamics during baseline light and dark phase recordings using two-way ANOVA with genotype (group 1: matDp/ + , WT; group 2: patDp/ + , WT; group 3: Ube3a, WT) and time (2-h bins) as factors, and average beta power across time using t test. Interactions reported for genotype x time

Frequency	State	Genotype	Interactions			
			Light phase		Dark phase	
Beta	Wake	matDp/ +	F (5, 75) = 0.5442	*P* = 0.7422	F (5, 75) = 0.4737	*P* = 0.7947
		patDp/ +	F (5, 30) = 0.5573	*P* = 0.7316	F (5, 30) = 0.6159	*P* = 0.6886
		Ube3a	F (5, 30) = 2.115	*P* = 0.0910	F (5, 30) = 0.2150	*P* = 0.9534
	NREM	matDp/ +	F (5, 75) = 0.4544	*P* = 0.8088	F (5, 75) = 1.250	*P* = 0.2945
		patDp/ +	F (5, 30) = 1.073	*P* = 0.3948	F (5, 30) = 0.6643	*P* = 0.6533
		Ube3a	F (5, 30) = 1.383	*P* = 0.2587	F (5, 30) = 0.5622	*P* = 0.7281
	REM	matDp/ +	F (5, 75) = 0.6822	*P* = 0.6384	F (5, 75) = 0.4315	*P* = 0.8253
		patDp/ +	F (5, 30) = 0.3498	*P* = 0.8783	F (5, 30) = 1.346	*P* = 0.2723
		Ube3a	F (5, 30) = 1.288	*P* = 0.2951	F (5, 30) = 1.345	*P* = 0.2729
Beta_average	Wake	matDp/ +	t = 2.145, df = 15	***P* = 0.0487**	t = 2.168, df = 15	***P* = 0.0467**
	NREM	matDp/ +	t = 3.536, df = 15	***P* = 0.0030**	t = 3.337, df = 15	***P* = 0.0045**
	REM	matDp/ +	t = 2.882, df = 15	***P* = 0.0114**	t = 2.724, df = 15	***P* = 0.0157**
Delta	Wake	matDp/ +	F (5, 75) = 1.151	*P* = 0.3414	F (5, 75) = 1.502	*P* = 0.1994
		patDp/ +	F (5, 30) = 0.5903	*P* = 0.7073	F (5, 30) = 0.7578	*P* = 0.5872
		Ube3a	F (5, 30) = 1.734	*P* = 0.1572	F (5, 30) = 0.5635	*P* = 0.7271
	NREM	matDp/ +	F (5, 75) = 0.2177	*P* = 0.9539	F (5, 75) = 1.117	*P* = 0.3585
		patDp/ +	F (5, 30) = 0.9128	*P* = 0.4860	F (5, 30) = 0.5793	*P* = 0.7154
		Ube3a	F (5, 30) = 1.160	*P* = 0.3517	F (5, 30) = 0.7352	*P* = 0.6029
Theta	REM	matDp/ +	F (5, 75) = 0.8366	*P* = 0.5279	F (5, 75) = 0.3561	*P* = 0.8768
		patDp/ +	F (5, 30) = 0.5433	*P* = 0.7419	F (5, 30) = 0.1093	*P* = 0.9894
		Ube3a	F (5, 30) = 2.223	*P* = 0.0779	F (5, 30) = 1.619	*P* = 0.1854

### Recovery sleep and sleep fragmentation

**Fig. 5:**
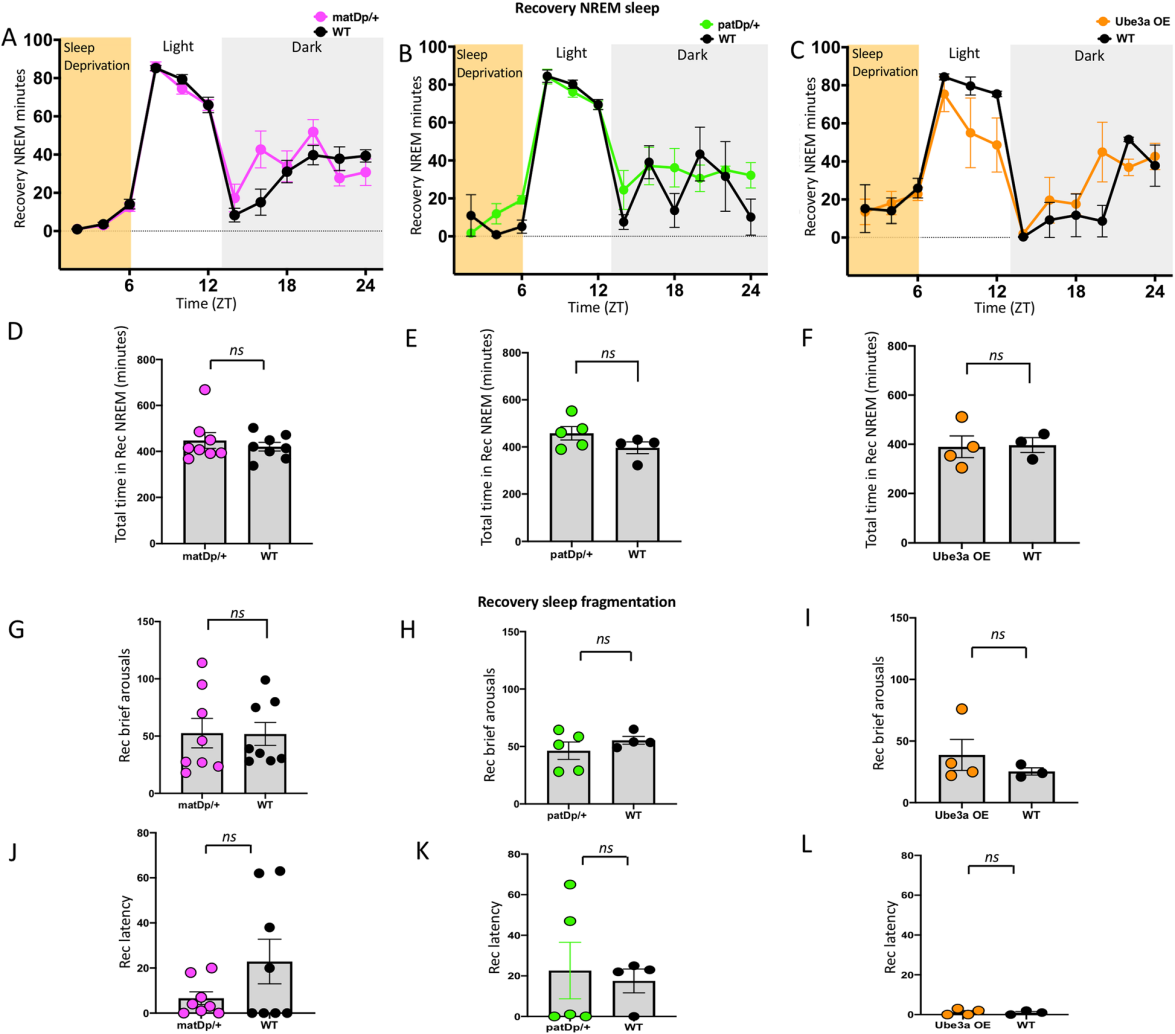
Sleep homeostasis in Dup15q mice. Temporal pattern of NREM sleep during 6 h of sleep deprivation, followed by an 18-h recovery sleep in matDp/ + (**A**), patDp/ + (**B**), and Ube3a OE (**C**) mice. The yellow-shaded area represents the time in sleep deprivation. Total NREM sleep time in matDp/ + (**D**), patDp/ + (**E**), and Ube3a OE (**F**) during recovery sleep. Number of brief arousals (**G**-**I**) and time to sleep onset (**J**-**L**) in matDp/ + , patDp/ + , and Ube3a OE groups compared with WT littermate controls during recovery sleep

In order to examine the homeostatic regulation of sleep in these mouse models, we examined NREM sleep rebound after 6 h of sleep deprivation. We recorded the temporal pattern of NREM sleep during 18 h of recovery sleep following 6 h of sleep deprivation and analyzed with genotype and time as factors using a two-way ANOVA. We found no significant differences between Dup15q mutant mice and their littermate controls: matDp/ + , F_(11, 154)_ = 1.311, *P* = 0.2378 (Fig. 5A); patDp/ + , F_(11, 77)_ = 1.300, *P* = 0.2407 (Fig. 5B); Ube3a OE, F_(11, 55)_ = 1.652, *P* = 0.1098 (Fig. 5C). The total amount of time spent in NREM sleep during recovery did not differ between mutants and controls in all three groups: matDp/ + , *P* = 0.5055, t = 0.6835, df = 14 (Fig. 5D); patDp/ + , *P* = 0.1602, t = 1.571, df = 7 (Fig. 5E); Ube3a OE, *P* = 0.9103, t = 0.1185, df = 5 (Fig. 5F). There were no differences in the number of brief arousals exhibited by mutant mice compared to littermate controls: matDp/ + , *P* = 0.9639, t = 0.0461, df = 14 (Fig. 5G); patDp/ + , *P* = 0.3504, t = 1.000, df = 7 (Fig. 5H); Ube3a OE, *P* = 0.4151, t = 0.8883, df = 5 (Fig. 5I). Time to sleep onset also did not differ in all three groups: matDp/ + , *P* = 0.1363, t = 1.581, df = 14 (Fig. 5J); patDp/ + , *P* = 0.7678, t = 0.3070, df = 7 (Fig. 5K); Ube3a OE, *P* = 0.8144, t = 0.2474, df = 5 (Fig. 5L). Therefore, there were no differences in the temporal patterns of the different sleep stages in Dup15q animals after sleep deprivation. Table 4 includes analysis of sleep waveforms during recovery after sleep deprivation, separated by light and dark cycles.

**Table 4:** Analysis of sleep waveforms in light and dark phase recordings during recovery after 6 h of sleep deprivation, using two-way ANOVA with genotype (group 1: matDp/ + , WT; group 2: patDp/ + , WT; group 3: Ube3a, WT) and time (2-h bins) as factors. Interactions reported for genotype x time

State	Genotype	Interactions			
		Light phase		Dark phase	
NREM	matDp/ +	F (5, 70) = 0.4493	*P* = 0.8124	F (5, 70) = 3.983	***P* = 0.0031**
	patDp/ +	F (5, 35) = 3.050	***P* = 0.0218**	F (5, 35) = 1.301	*P* = 0.2859
	Ube3a	F (5, 25) = 0.9946	*P* = 0.4411	F (5, 25) = 1.660	*P* = 0.1811
REM	matDp/ +	F (5, 70) = 0.6243	*P* = 0.6817	F (5, 70) = 2.047	*P* = 0.0826
	patDp/ +	F (5, 30) = 0.6690	*P* = 0.6499	F (5, 30) = 2.169	*P* = 0.0841
	Ube3a	F (5, 25) = 0.4616	*P* = 0.8009	F (5, 25) = 1.300	*P* = 0.2956

**Fig. 6:**
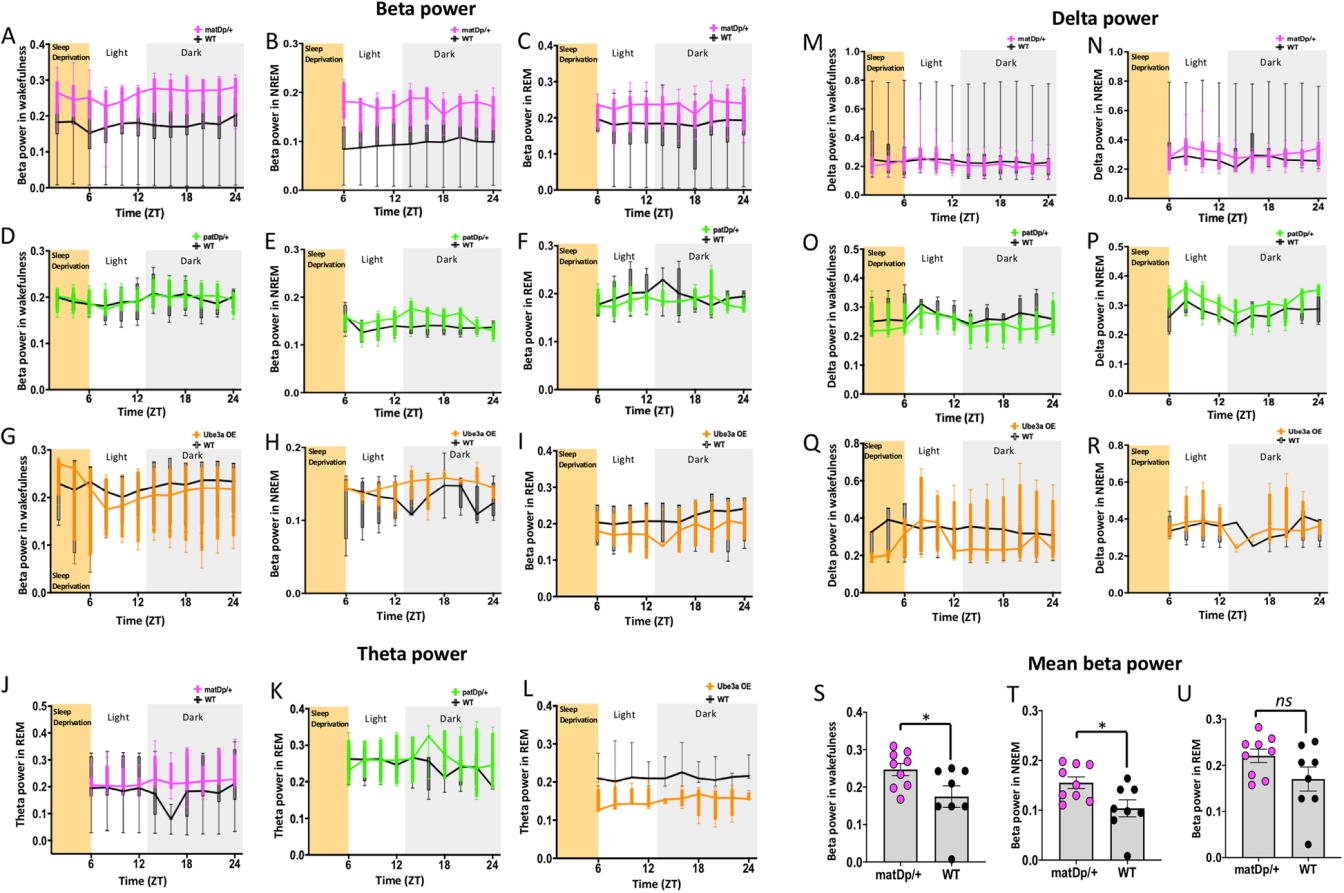
Spectral power dynamics during recovery after sleep deprivation. Beta power dynamics during wakefulness, NREM, and REM sleep in matDp/ + (**A**-**C**), patDp/ + (**D**-**F**), and Ube3a OE (**G**-**I**) groups compared to WT littermate controls. The yellow-shaded area represents time in sleep deprivation. Delta power dynamics during wakefulness and NREM sleep in matDp/ + (**J**, **M**), patDp/ + (**K**, **N**), and Ube3a OE (**L**, **O**) groups compared to WT littermate controls. Theta power dynamics during REM sleep in matDp/ + (**P**), patDp/ + (**Q**), and Ube3a OE (**R**) groups compared to WT littermate controls. Mean beta power calculated across the 18-h recovery recording compared between matDp/ + and WT littermate controls during wakefulness (**S**), NREM (**T**), and REM (**U**) sleep

Power spectral analysis of 18 h of continuous EEG recordings after the 6-h sleep deprivation revealed that mutant mice in the matDp/ + group continued to have elevated beta power during recovery (Fig. 6 A-C). Mean beta power calculated across the recording showed significantly elevated beta power in the matDp/ + group during wakefulness and NREM sleep but not in REM sleep: wakefulness, *P* = 0.0394, t = 2.256, df = 15 (Fig. 6S); NREM, *P* = 0.0222, t = 2.549, df = 15 (Fig. 6T); REM, *P* = 0.1042, t = 1.730, df = 15 (Fig. 6U). Beta power, however, did not differ between mutant mice and WT controls in both patDp/ + : wakefulness, F_(11, 66)_ = 0.6762, *P* = 0.7558 (Fig. 6D); NREM, F_(9,54)_ = 1.481, *P* = 0.1189 (Fig. 4E); REM, F_(9,54)_ = 0.8482, *P* = 0.5758 (Fig. 6F), and the Ube3a OE: wakefulness, F_(11, 66)_ = 0.4448, *P* = 0.9295 (Fig. 6G); NREM, F_(9,54)_ = 1.188, *P* = 0.3218 (Fig. 6H); REM, F_(9,54)_ = 0.8458, *P* = 0.5779 (Fig. 6I) groups. Theta power during REM sleep did not differ between Dup15q mice and WT controls in all three groups: matDp/ + , F_(9,117)_ = 2.131, *P* = 0.321 (Fig. 6J); patDp/ + , F_(9,54)_ = 1.343, *P* = 0.2373 (Fig. 6K); Ube3a OE, F_(9,45)_ = 0.2832, *P* = 0.9760 (Fig. 6L). Delta power did not differ in all three groups either during wakefulness: matDp/ + , F_(11, 165)_ = 0.8347, *P* = 0.6056 (Fig. 6M); patDp/ + , F_(11, 66)_ = 0.5054, *P* = 0.8930 (Fig. 6O); Ube3a OE, F_(11, 66)_ = 1.419, *P* = 0.1857 (Fig. 6Q), or during NREM sleep: matDp/ + , F_(9,135)_ = 1.552, *P* = 0.1359 (Fig. 6N); patDp/ + , F_(9,54)_ = 1.354, *P* = 0.2319 (Fig. 6P); Ube3a OE, F_(9,54)_ = 0.9002, *P* = 0.5317 (Fig. 6R). Therefore, similar to that seen in baseline recording, elevated beta power in matDp/ + persisted during recovery sleep, across sleep and wake stages. Table 5 includes analysis of power spectral dynamics during recovery after sleep deprivation, separated by light and dark cycles.

**Table 5:** Analysis of power spectral dynamics in light and dark phase recordings during recovery after 6 h of sleep deprivation, using two-way ANOVA with genotype (group 1: matDp/ + , WT; group 2: patDp/ + , WT; group 3: Ube3a, WT) and time (2-h bins) as factors, and average beta power across time using t test. Interactions reported for genotype x time

Frequency	State	Genotype	Interactions			
			Light phase		Dark phase	
Beta	Wake	matDp/ +	F (5, 75) = 0.8756	*P* = 0.5017	F (5, 75) = 0.7173	*P* = 0.6124
		patDp/ +	F (5, 30) = 0.09704	*P* = 0.9919	F (5, 30) = 1.581	*P* = 0.1957
		Ube3a	F (5, 30) = 0.6072	*P* = 0.6949	F (5, 30) = 0.4081	*P* = 0.8393
	NREM	matDp/ +	F (3, 45) = 11.42	***P* < 0.0001**	F (5, 75) = 3.256	***P* = 0.0103**
		patDp/ +	F (3, 18) = 2.404	*P* = 0.1011	F (5, 30) = 2.402	*P* = 0.0603
		Ube3a	F (3, 18) = 0.7913	*P* = 0.5145	F (5, 30) = 1.526	*P* = 0.2114
	REM	matDp/ +	F (3, 45) = 0.8003	*P* = 0.5003	F (5, 75) = 0.8321	*P* = 0.5310
		patDp/ +	F (3, 18) = 1.231	*P* = 0.3276	F (5, 30) = 0.8958	*P* = 0.4965
		Ube3a	F (3, 18) = 0.4818	*P* = 0.6991	F (5, 30) = 1.838	*P* = 0.1355
Beta_average	Wake	matDp/ +	t = 2.464, df = 13	***P* = 0.0284**	t = 1.649, df = 14	*P* = 0.1215
	NREM	matDp/ +	t = 2.183, df = 13	***P* = 0.048**	t = 2.215, df = 13,	***P* = 0.0452**
	REM	matDp/ +	t = 1.290, df = 14	*P* = 0.2179	t = 1.082, df = 14	*P* = 0.2976
Delta	Wake	matDp/ +	F (5, 75) = 1.099	*P* = 0.3684	F (5, 75) = 0.2749	*P* = 0.9255
		patDp/ +	F (5, 30) = 0.04741	*P* = 0.9985	F (5, 30) = 1.660	*P* = 0.1749
		Ube3a	F (5, 30) = 2.283	*P* = 0.0715	F (5, 30) = 1.725	*P* = 0.1592
	NREM	matDp/ +	F (3, 45) = 2.574	*P* = 0.2111	F (5, 75) = 1.583	*P* = 0.1753
		patDp/ +	F (3, 18) = 0.7020	*P* = 0.5631	F (5, 30) = 2.298	*P* = 0.0700
		Ube3a	F (3, 18) = 0.9471	*P* = 0.4387	F (5, 30) = 1.657	*P* = 0.1756
Theta	REM	matDp/ +	F (3, 39) = 2.368	*P* = 0.0855	F (5, 65) = 1.091	*P* = 0.3741
		patDp/ +	F (3, 18) = 1.149	*P* = 0.3563	F (5, 30) = 0.7484	*P* = 0.5937
		Ube3a	F (3, 15) = 0.8304	*P* = 0.4977	F (5, 25) = 0.2709	*P* = 0.9247

## Discussion

Sleep is a fundamental physiological process that plays a pivotal role in various aspects of neurodevelopment and overall well-being [2, 39–41]. It is during sleep that critical functions such as memory consolidation [42–44], neural plasticity [45–47], and emotional regulation [40, 48–51] take place. Individuals with Dup15q syndrome experience disrupted sleep, characterized by abnormal NREM sleep [10] and elevated beta oscillations [10, 12, 13]. The exact mechanisms driving these disturbances remain unknown. These sleep disruptions can reduce sleep quality, increase daytime sleepiness, and cause cognitive impairments, all of which are commonly observed in individuals with Dup15q syndrome [7, 8, 12, 19, 38]. Concurrently, elevated beta oscillations during sleep and wakefulness can disrupt neural synchronizations and communication, potentially contributing to difficulties in maintaining stable sleep patterns as well as sleep-dependent neural plasticity processes.

To ascertain the feasibility of modeling these sleep disturbances in mice and to pinpoint the specific genetic factors involved, we quantified sleep parameters (baseline sleep, sleep fragmentation, spectral power dynamics during different sleep states, and recovery following sleep deprivation) in chromosome-engineered mice with maternal (matDp/ +) or paternal (patDp/ +) inheritance of the full 15q11.2-13.1 duplication [14], and mice with overexpression of the UBE3A gene (Ube3a OE) in the 15q11.2-13.1 duplication region, resulting in one extra copy of Ube3a [15]. We found no differences in the ability of the three strains of Dup15q mice to cycle through different sleep stages, and no differences in sleep duration between these Dup15q mice and WT controls. Sleep fragmentation patterns remained comparable across both groups, while matDp/ + and Ube3a OE mice exhibited significantly reduced time to sleep onset after light onset. Notably, in the matDp/ + mice, beta oscillations were elevated across all brain states (wakefulness, NREM, and REM) during baseline recording, consistent with the characteristic beta oscillation patterns observed in the Dup15q clinical population. Beta oscillations also continued to be elevated in wakefulness and NREM during recovery recordings after a 6-h sleep deprivation. However, Dup15q mice and their control counterparts exhibited similar patterns of recovery NREM sleep following sleep deprivation. Overall, the findings in this study shed light on several important aspects of sleep and neural activity in Dup15q mice, highlighting both similarities and differences between these animal models and the human condition.

### Intact sleep architecture in Dup15q mice

The ultradian sleep cycle, characterized by the episodic progression through NREM and REM sleep, is a fundamental aspect of sleep physiology closely linked to cognitive and neural processes [52, 53]. Disrupted ultradian cycling of sleep can have profound implications for individuals with NDDs, as it may perturb the finely tuned processes driving memory consolidation, neural plasticity, and cognitive function. Our study revealed no discernible differences in the distribution of sleep stages, encompassing NREM and REM sleep when comparing Dup15q mice to their WT littermate controls across both the light and dark phases (Fig. 2A). This suggests that the fundamental capability to transition through sleep stages remains unaltered in Dup15q mice. Additionally, sleep duration during NREM and REM sleep did not differ between mutants and controls across the light and dark phases in all three groups (Fig. 3A-L). This finding is not consistent with the strongly reduced NREM sleep seen in Dup15q individuals[10], indicating that some aspects of altered sleep physiology in Dup15q individuals cannot be successfully modeled in these three animal models. This could result from compensatory mechanisms in mice, less severe overexpression of Dup15q11-13 genes in these animals, or evolutionary specializations controlling NREM sleep patterns that are not present in mice. Clearly, intricate regulatory machinery governing transitions between sleep stages remains intact in Dup15q mice.

### Reduced time to sleep onset in Dup15q mice

Given the reported sleep disturbances, both behavioral [7]and physiological [6, 10], in individuals with Dup15q syndrome, we evaluated sleep parameters that reflected the degree of disruption in sleep architecture in Dup15q mice. During the 24-h baseline sleep, at the level of brief arousals, which reflect transient disturbances during sleep, the overall quality of sleep remained comparable between Dup15q mice and WT controls in all three groups. However, time to sleep onset was significantly reduced in the matDp/ + and Ube3a OE groups compared to their WT controls. Time to sleep onset reflects the time it takes for an individual to transition from wakefulness to sleep. In contrast to these findings, studies have shown that individuals with ASD [54–56] and attention deficit hyperactivity disorder (ADHD) [47, 57–60] have increased time to sleep onset, greater number of awakenings during sleep, and a general lower sleep quality compared with healthy individuals, with implications on their overall development and cognition. The observed reduction in time to sleep onset in Dup15q mice may be linked to physiologic sleepiness, which may be influenced by factors such as homeostatic sleep drive and circadian rhythms. This could involve changes in neural circuits, that influence the initiation of sleep.

After 6-h of sleep deprivation, WT mice exhibited reduced time to sleep onset compared to their baseline values. This aligns with expectations reflecting a compensatory increase in sleep drive following sleep deprivation. This phenomenon is consistent with the homeostatic regulation of sleep, where extended wakefulness leads to an accumulation of sleep pressure [61–65]. However, the notable finding that Dup15q mice exhibit reduced time to sleep onset even under baseline sleep conditions without prior sleep deprivation suggests that the mechanisms governing sleep initiation are already altered in Dup15q mice, independent of acute sleep loss. The reduced time to sleep onset in both baseline and recovery sleep in Dup15q mice underscores a fundamental shift in sleep dynamics and raises questions about the specific neural and molecular factors contributing to this altered sleep phenotype in Dup15q mice.

### Elevated beta oscillations in Dup15q mice

The deviations in sleep onset in Dup15q mice prompted us to examine the spectral dynamics that organize and synchronize neural activity during various states of consciousness. Moreover, prior studies have shown that in addition to having abnormal NREM sleep, individuals with Dup15q syndrome have increased beta oscillations during awake resting state [11–13, 38] and during sleep [6, 10]. Here, we found that matDp/ + mice showed increased beta oscillation power across all brain states (wakefulness, NREM, and REM sleep). This pervasive elevation of beta oscillations, in matDp/ + , while not seen in patDp/ + or UBE3a OE mice, mirrors that seen in humans with Dup15q. This suggests that the genetic imprinting of the critical 15q11.2–13.1 region has a significant impact on neural activity patterns. This implies that alterations in the UBE3A gene, its subsequent expression, and its interactions with other overexpressed genes in the critical region, particularly significant in maternally inherited Dup15q mice, play a unique role in modulating neural oscillations during different sleep states. Moreover, the elevated beta power in matDp/ + group persists during recovery sleep after the 6-h sleep deprivation. This indicates that not only does the beta EEG biomarker not change with brain-state, but it does not change with processes of sleep homeostasis, making it a reliable biomarker of disease. In contrast with our findings in the patDp/ + group, two individuals with paternal duplication of the 15q11.2-13.1 region have been shown to have elevated beta oscillations [38]. This difference between mouse and human sleep physiology may reflect the limitations of disease modeling in these animals.

Beta oscillations, typically associated with active wakefulness, motor planning, and sensory planning, play a crucial role in facilitating efficient neural communication and synchronization [66–70]. When these oscillations become heightened, particularly during sleep, their consequences may extend to disruptions in memory consolidation, emotional regulation, and overall cognitive performance. Beta oscillations during sleep may be intricately linked to processes such as synaptic plasticity and memory encoding [71, 72]. Therefore, their aberrant elevation may interfere with these critical functions, potentially contributing to the cognitive impairments often observed in NDDs. Moreover, elevated beta oscillations may disrupt the transitions between different sleep stages, affecting the balance between restorative REM sleep and deep NREM sleep. This disturbance in sleep architecture can lead to increased daytime sleepiness, impaired attention, and heightened susceptibility to mood disorders. Therefore, elevated beta oscillations in sleep may have far-reaching consequences not only for sleep quality but also for overall cognitive development in Dup15q syndrome. The identification of increased beta oscillations in Dup15q mice, mirroring findings in humans, is particularly exciting as it provides a robust translatable biomarker in this murine model for investigating the neural underpinnings of sleep disturbances in this disorder. Understanding the functional ramifications of the beta biomarker is pivotal for developing targeted therapeutic interventions aimed at ameliorating sleep-related challenges and cognitive impairments in affected individuals.

In contrast to the profound reduction of delta oscillations observed in individuals with Dup15q syndrome [10, 12], we found no difference in delta oscillation power in Dup15q mice. Delta oscillations are crucial for the restorative functions of deep sleep, including memory consolidation and overall cognitive rejuvenation and reflect the homeostatic mechanisms underlying sleep organization [73–76]. The preservation of delta oscillations in Dup15q mice suggests a divergence from the clinical presentation in humans and underscores the importance of species-species considerations in translational research. Moreover, theta oscillations, associated with memory processes and neural integration [77, 78], remain unchanged in Dup15q mice. The interplay of these spectral dynamics in Dup15q mice not only inform our understanding of sleep disturbances in the context of this NDD but also open new avenues for investigations that consider the intricacies of neural activity during different sleep states.

### Preserved homeostatic mechanisms in Dup15q mice

Sleep homeostasis is a fundamental regulatory mechanism that ensures a balance between sleep and wakefulness, adjusting based on prior sleep duration and quality [61, 65, 79]. The unaltered recovery sleep in Dup15q mice suggests that, despite the genetic alterations associated with the disorder, the capacity to respond to sleep loss and compensate with extended recovery sleep remains intact. This finding is significant as it implies a level of resilience in Dup15q mice in the fundamental processes governing sleep-wake regulation. The specific disruptions in sleep patterns observed in Dup15q syndrome may be more related to intrinsic sleep regulatory processes rather than the ability to recover from sleep debt. It is, therefore, essential to identify the precise molecular and neural components responsible for this homeostatic control to further our understanding of the sleep disturbances in Dup15q syndrome.

## Conclusions and future directions

In conclusion, our investigation into sleep physiology in Dup15q mice has revealed disrupted neural synchronization marked by elevated beta oscillations in matDp/ + mice, during both baseline and recovery sleep, potentially contributing to the sleep disturbances observed in the clinical population. Despite the anticipated changes in NREM sleep and sleep homeostasis, recovery following sleep deprivation remains unaltered, suggesting a unique resilience. While the reduced time to sleep onset observed in Dup15q mice hints at a specific facet of sleep initiation challenges in these models, the altered spectral dynamics, with elevated beta oscillations, reveal a broader disruption in neural coordination across wakefulness and sleep states. The convergence of these findings emphasizes the multifaceted nature of sleep disturbances in Dup15q syndrome. These findings lay a foundation for further investigations to scrutinize the specific neural circuits, molecular pathways, and potential compensatory mechanisms at play in Dup15q mice, enhancing our understanding of the nuanced relationship between sleep disturbances and NDDs like Dup15q syndrome. Ultimately, this knowledge is indispensable for refining the translational relevance of murine models in studying sleep disorders and for developing targeted interventions that can improve the quality of life for affected individuals.

## Limitations

While our study provides valuable insights into sleep dynamics in Dup15q mice, several limitations warrant consideration. Translating findings from mouse models to humans presents a significant challenge due to inherent differences in neurobiology and behavior. While murine models offer valuable insights, behavioral and neurophysiological responses to sleep-related disruptions, as well as the genetic and molecular underpinnings of NDDs, may not precisely mirror the intricacies observed in affected human populations. Furthermore, the study’s scope is limited to sleep-related parameters, and a broader exploration of neurobehavioral aspects and neurophysiological mechanisms associated with the sleep disturbances in Dup15q syndrome, would be valuable. Investigating specific circuit motifs and neuronal networks implicated in sleep regulation, particularly those uniquely altered in Dup15q syndrome, could offer a more comprehensive understanding of the neural underpinnings of disrupted sleep in this condition. Overall, these mouse models of Dup15q syndrome are promising tools to reveal potential mechanisms and biomarkers that underlie the disease and its phenotypes. Future research should take these into consideration and aim for a comprehensive approach that incorporates both murine models and human clinical data to bridge the translational gap effectively.

## Data Availability

The data sets used in this study are available from the corresponding author on reasonable request.

## Abbreviations

Dup15q syndrome: Duplications of 15q11.2-13.1
NDD: Neurodevelopmental disorder
GABA_A_R: Gamma-aminobutyric acid type A receptor
EEG: Electroencephalogram
NREM: Non-rapid eye movement sleep
REM: Rapid eye movement sleep
ASD: Autism spectrum disorder
ID: Intellectual disability
NIH: National Institute of Health
ANOVA: Analysis of Variance
UCLA: University of California, Los Angeles
